# Assessing the clinical utility of genomic expression data across human cancers

**DOI:** 10.18632/oncotarget.10002

**Published:** 2016-06-14

**Authors:** Xinsen Xu, Lei Huang, Chun Hei Chan, Tao Yu, Runchen Miao, Chang Liu

**Affiliations:** ^1^ Department of Hepatobiliary Surgery, The First Affiliated Hospital of Xi'an Jiaotong University, Xi'an, China; ^2^ 118 Lancaster Terrace, Brookline, MA, USA; ^3^ Faculty of Medicine, The Chinese University of Hong Kong, Hong Kong; ^4^ Department of Neurosurgery, Peking University First Hospital, Beijing, China

**Keywords:** genome, cancer, expression, utility, prognosis

## Abstract

Cancer molecular profiling provides better understanding of tumor mechanisms and helps to improve the existing cancer management. Here we present the gene expression signatures from ∼9000 human tumors with clinical information across 32 malignancies from The Cancer Genome Atlas project (TCGA). Major predictors from the RNA sequencing data that were significantly correlated with cancer survival were identified. The expression level of these prognostic genes revealed significant genomic pathways that were clinically relevant to survival outcomes across human cancers. Furthermore, it is shown that in most cancer types, combinations of these genomic signatures with clinical information might yield improved predictions. Thus, with respect to clinical utility, our study reveals the promising values of genomic data from the pan-cancer perspective.

## INTRODUCTION

Cancer is a global health burden and the second leading cause of death [1]. Despite various detection method and treatment options, survival rates for most cancers are still very low.

Genomic features emerge as promising biomarkers for cancer [2]. Of the various molecular data, the gene expression value from RNA sequencing revealed detailed molecular features with prognostic associations [3]. However, due to high cost, most studies only focused on several pre-selected genes, or based on small sample sizes [4, 5].

The cancer genome atlas (TCGA) project “motivated large-scale genomic efforts to obtain the complete catalogs of the genomic alterations in cancer” [6]. Besides the rich molecular features (genomic, transcriptomic, epigenomic and proteomic) of each tumor, it also provides valuable clinical information. However, the clinical utility of these data has not been fully elucidated.

In the present study, we depicted the global pan-cancer prognostic landscape by analyzing the expression signatures from ~9000 human tumors across 32 malignancies from TCGA data sets. Furthermore, the clinical utility of survival predictions was evaluated by combining the genomic data with clinical information.

## RESULTS

### Patient characteristics and outcome

Patient information with complete RNA sequencing data and clinical data of all TCGA cancer types were collected (32 tumor types) (adrenocortical carcinoma, ACC; bladder urothelial carcinoma, BLCA; breast invasive carcinoma, BRCA; cervical and endocervical cancers, CESC; cholangiocarcinoma, CHOL; colon adenocarcinoma, COAD; lymphoid neoplasm diffuse large B-cell lymphoma, DLBC; esophageal carcinoma, ESCA; glioma, GBMLGG; head and neck squamous cell carcinoma, HNSC; kidney chromophobe, KICH; kidney renal clear cell carcinoma, KIRC; kidney renal papillary cell carcinoma, KIRP; acute myeloid leukemia, LAML; liver hepatocellular carcinoma, LIHC; lung adenocarcinoma, LUAD; lung squamous cell carcinoma, LUSC; mesothelioma, MESO; ovarian serous cystadenocarcinoma, OV; pancreatic adenocarcinoma, PAAD; pheochromocytoma and paraganglioma, PCPG; prostate adenocarcinoma, PRAD; rectum adenocarcinoma, READ; sarcoma, SARC; skin cutaneous melanoma, SKCM; stomach adenocarcinoma, STAD; testicular germ cell tumors, TGCT; thyroid carcinoma, THCA; thymoma, THYM; uterine corpus endometrial carcinoma, UCEC; uterine carcinosarcoma, UCS; uveal melanoma, UVM).

**Figure 1:**
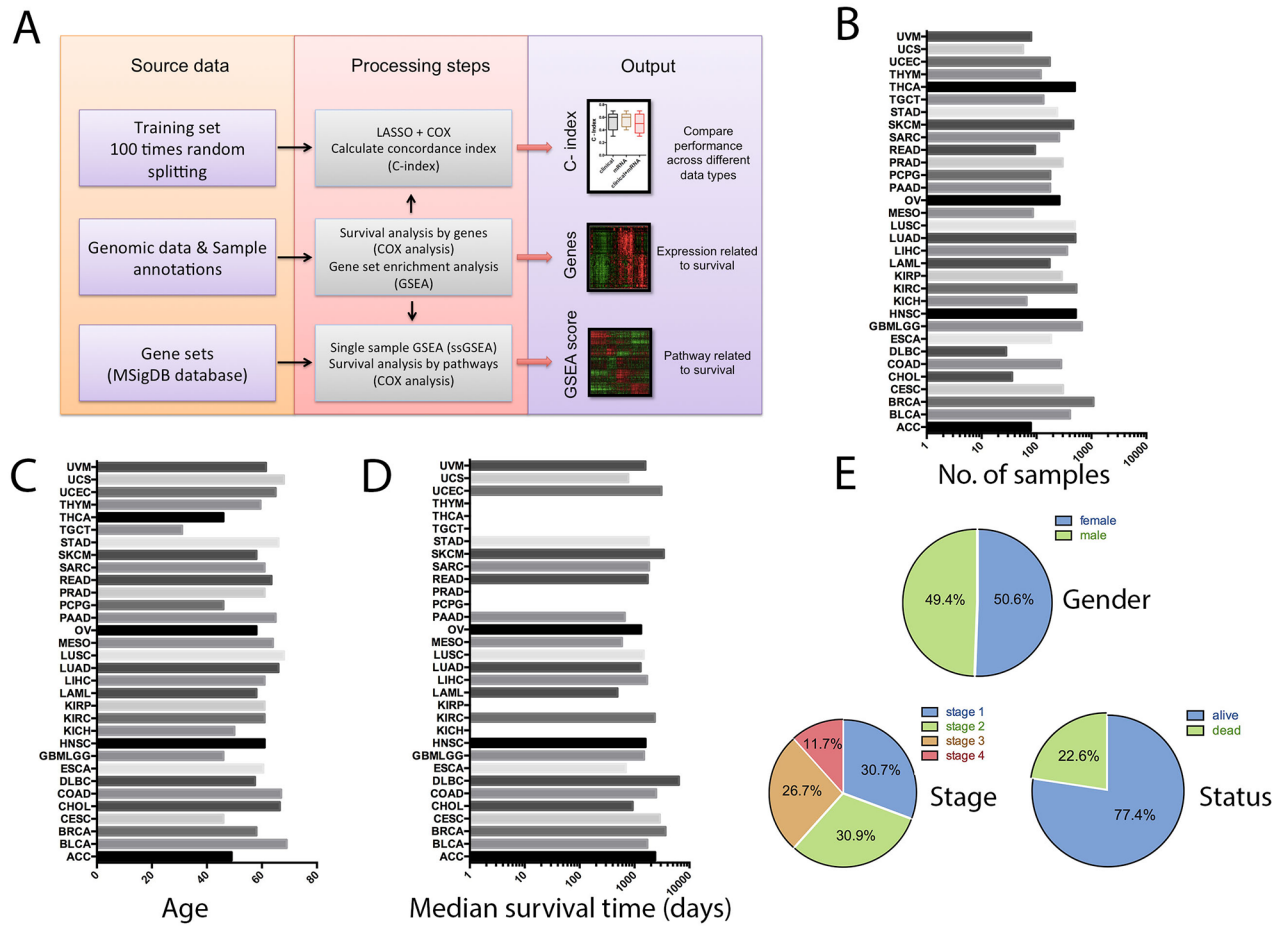
Overview of the computational approach and patient characteristics **A.** Flow diagram summarizing the data processing and analysis steps. **B.** Number of patient samples with survival data, organized by cancer types. **C.** Median age of the patients in different cancer types. **D.** Median survival time of the patients in different cancer types (some of the cancer types don't have enough death events to calculate the median survival times, either because of the high survival rates or due to the small sample size of the cancer type). **E.** Frequency distributions of gender, tumor stage and survival outcome in the whole cancer population.

Data analysis steps and clinical characteristics of the cancer patients were shown in Figure 1A-1E. In total, there were 9175 patients of the 32 tumor types, in which, 49.4% were male and 50.6% were female. Their median age was 60 years old. With respect to tumor stage, 30.7% of the patients were in stage 1, 30.9% were in stage 2, 26.7% were in stage 3, and 11.7% were in stage 4. At the time of analysis, 77.4% of the patients remained alive, and 22.6% were deceased.

### Pan-cancer prognostic genes and risk scores

**Figure 2:**
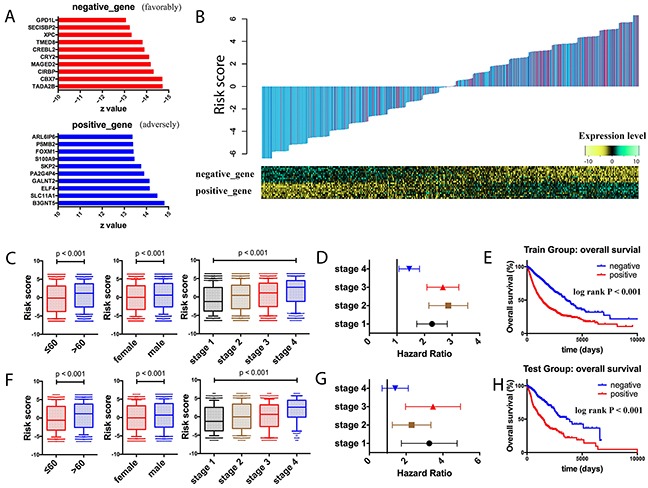
Prognostic landscape of gene expression in the whole cancer population **A.** Top ten adverse and favorable pan-cancer prognostic genes were identified in the training group, ranked by the z scores. **B.** Risk score calculated by the top prognostic genes in the training group patients. Upper panel: risk-score distribution of the training group patients and survival status (blue indicates alive, and red indicates dead). Lower panel: heatmap showing the expression level of the top prognostic genes. **C.** Box plots of risk scores in different age groups, different gender groups, and different stage groups in the training group patients. **D.** Forest plot of risk score association with cancer mortality in the training group patients of different stages. **E.** Kaplan-Meier estimates of overall survival according to the risk score in the training set. **F.** Box plots of risk scores in different age groups, different gender groups, and different stage groups in the testing group patients. **G.** Forest plot of risk score association with cancer mortality in the testing group patients of different stages. **H.** Kaplan-Meier estimates of overall survival according to the risk score in the testing set.

To explore the pan-cancer prognostic signatures, pan-cancer dataset was built by combining all the cancer patients. Samples were randomly assigned into two groups, where 80% of the samples were assigned as the training group and 20% as the testing group. By cox regression analysis for the training group, the top ten adverse genes (B3GNT5, SLC11A1, ELF4, GALNT2, PA2G4P4, SKP2, S100A9, FOXM1, PSMB2, ARL6IP6) and top ten favorable prognostic genes (TADA2B, CBX7, CIRBP, MAGED2, CRY2, CREBL2, TMED8, XPC, SECISBP2, GPD1L) were identified (Figure 2A). Based on these top prognostic genes, risk scores were calculated (Table 1). The risk score was defined as the weighted sums of the independent prognostic gene values (1 for high expression, and 0 for low expression), weighted with their regression coefficients from the cox models (Figure 2B).

**Table 1:** Specific risk scores for different types of cancer

Cancer Type	Risk Score
Whole population (binary)	Score = 0.66*B3GNT5+0.65*SLC11A1+0.65*ELF4+0.65*GALNT2+0.63*PA2G4P4+0.63*SKP2+0.60*S100A9+0.63*FOXM1+0.61*PSMB2+0.64*ARL6IP6-0.63*GPD1L-0.62*SECISBP2-0.58*XPC-0.61*TMED8-0.61*CREBL2-0.64*CRY2-0.64*MAGED2-0.68*CIRBP-0.69*CBX7-0.71*TADA2B
Whole population(continuous)	Score = 0.09*B3GNT5+0.17*SLC11A1+0.29*ELF4+0.12*GALNT2+0.18*PA2G4P4+0.16*SKP2+0.08*S100A9+0.14*FOXM1+0.04*PSMB3+0.23*ARL6IP6-0.34*GPD1L-0.35*SECISBP2-0.38*XPC-0.26*TMED8-0.36*CREBL2-0.37*CRY2-0.46*MAGED2-0.54*CIRBP-0.48*CBX7-0.42*TADA2B
ACC	Score = 2.48*MASTL+2.38*RECQL4+2.33*PRC1+2.36*KIF11+2.65*AMMECR1L+2.55*TRIP13+2.54*MKI67+2.59*NCAPD3+2.07*E2F1+2.06*FANCI-2.07*APH1B-2.3*CTSA-2.04*UPRT-2.02*HNRNPH2-2.28*NDRG4-2.47*PPFIBP2-1.94*LACTB-2.43*PTGR2-2.11*CHIC1-2.36*BDH2
BLCA	Score = 0.81*SPNS1+0.77*GARS+0.78*NBAS+0.77*IFT122+0.75*NOMO1+0.74*TMX2+0.73*DHRS4+0.74*CCDC28B+0.74*TMEM109+0.74*DAD1-0.86*GATA2-0.77*TRIM26-0.76*MRPS6-0.77*YDJC-0.76*ZNF841-0.75*ZBTB49-0.72*ORMDL1-0.72*DEDD2-0.72*OGT-0.74*CTSH
BRCA	Score = 0.88*ZHX1+0.82*PRRC1+0.82*SCRN1+0.81*IARS+0.81*PTPN11+0.83*VPS35+0.78*MRS2+0.77*GRPEL2+0.79*TMEM65+0.76*PGK1-0.95*TNFRSF14-0.95*KDM4B-0.92*INO80B-0.88*LOC150776-0.86*MRPL23-0.87*PYCARD-0.84*ABHD14A-0.82*FGD3-0.84*SEC14L2-0.79*NFKBIA
CESC	Score = 1.14*PHRF1+1.09*TNRC18+1.06*ITGA5+0.98*DBN1+1.01*LATS2+1.01*TOR1AIP2+1.02*FASN+1*URGCP+0.95*SRI+0.95*ADAM9-1.28*TREX1-1.21*RBM38-1.07*LGALS9-1.04*HNRNPA3-0.97*NQO2-0.96*ZER1-0.97*ISCU-0.94*MTCP1NB-0.96*AKR1A1-0.98*SLC25A28
CHOL	Score = 1.91*EIF5A+2*CEBPB+1.9*SCO1+1.89*ROM1+2.32*SRI+1.69*FAM54B+1.62*MNAT1+1.58*PSEN1+1.51*PDHB+1.66*SLC38A6-1.63*SCRN1-1.95*PGPEP1-1.83*EIF4ENIF1-1.62*SGSH-1.63*VSIG10-1.49*ACBD5-1.47*PURB-1.61*TNFAIP8-1.57*FUT4-1.38*FGD6
COAD	Score = 1.24*TIAL1+1.14*SMNDC1+1.1*KIAA0907+1.09*POLR2J4+1.03*HSPA1L+0.94*ZBTB25+0.95*UBN2+0.95*SCRN3+0.98*ZBTB9+0.93*DNAJB6-0.99*CPT2-1.01*MRPL37-0.99*ATP8B1-0.96*CCDC149-0.92*EIF2C1-0.9*DYNLL2-0.96*ZCCHC11-0.91*MFN2-1.01*GSR-0.9*SAMM50
DLBC	Score = 1.67*ELP4+1.48*API5+1.48*ARHGEF7+1.48*ATXN7L2+1.48*EXOC5+1.48*GMEB1+1.48*MEMO1+1.48*MPHOSPH10+1.48*MTOR+1.48*NEO1-1.48*TBKBP1-1.48*STXBP2-1.48*PUS1-1.48*PTRH1-1.48*POLR3D-1.48*KCNK6-1.48*IFI35-1.48*GPAA1-1.48*FHL3-1.48*FBXW5
ESCA	Score = 1.13*B3GALTL+1.11*PGK1+1.18*GRPEL2+1.17*MAPRE1+1.03*SRXN1+1.02*LRRC58+0.99*NFATC3+0.96*ST13+0.94*TRMT6+0.92*MLLT11-1.02*UNC13D-0.98*PCSK7-0.94*PLCD3-1.01*DIP2A-0.98*PLEKHM1P-0.89*UNC93B1-0.87*ERAP2-0.84*LRCH4-0.86*CCBL2-0.84*C10orf54
GBMLGG	Score = 1.98*GLA+1.83*KDELC2+2.01*WEE1+1.88*EMP3+1.8*DUSP10+1.84*CLIC1+1.88*TIMP1+1.84*CD58+1.79*DDB2+1.81*SHISA5-2.01*ZRANB1-1.9*GLUD1-1.88*FAM190B-1.78*RAP2A-1.79*ADD1-1.77*HDAC4-1.83*ARL3-1.74*PATZ1-1.79*SCAPER-1.73*RPL7
HNSC	Score = 0.9*PGK1+0.8*USP10+0.78*TOMM34+0.8*SNX6+0.72*TMED2+0.7*PDIA3P+0.69*ADK+0.71*USP14+0.69*TRIM32+0.68*HPRT1-0.75*ZNF266-0.69*ZNF700-0.64*AHCYL2-0.65*SH3BP2-0.65*ZNF577-0.64*ZNF557-0.64*ATXN7L2-0.64*ZNF20-0.63*DUSP16-0.63*CDK3
KICH	Score = 2.36*PNPT1+2.33*PTP4A2+2.31*GPN1+2.31*GPATCH2+2.3*PLEKHA2+2.29*NRAS+2.27*PDS5A+2.27*KDM1B+2.27*TTF2+2.26*NT5DC3-2.36*FIZ1-2.34*TST-2.34*C14orf1-2.34*ELAVL1-2.33*KLHL26-2.31*CES2-2.31*CTDP1-2.31*SUSD1-2.3*USF2-2.3*COPS7A
KIRC	Score = 1.28*DONSON+1.24*STRADA+1.2*ATP13A1+1.19*NOP56+1.18*CARS+1.18*ANAPC7+1.16*ANAPC5+1.14*SBNO2+1.15*NCLN+1.18*FKBP11-1.16*SGCB-1.15*PINK1-1.12*FBXO3-1.11*SSFA2-1.1*ITGA6-1.01*HBP1-1*FBXL3-1.02*RNF20-1*PURA-0.98*FBXL5
KIRP	Score = 1.7*GLT25D1+1.48*LMNB2+1.54*SPAG5+1.96*ADA+1.41*PUS7+1.61*CCNF+1.5*RHBDF2+1.7*P4HB+1.58*TSEN15+1.41*AEBP1-1.65*TMCO4-1.49*PGPEP1-1.63*FBXL5-1.51*HTATSF1-1.56*CCDC71-1.56*ACTR8-1.36*CC2D2A-1.42*PARP3-1.39*ZBTB3-1.39*SLC25A11
LAML	Score = 1.1*TOMM40L+0.95*NUP210+0.91*PARP3+0.83*DDIT4+0.83*CLCN5+0.79*FIBP+0.78*RPS6KA1+0.77*PSMA7+0.76*RINL+0.76*PARVB-0.99*PWWP2A-0.97*MBTPS1-0.87*NHLRC3-0.87*LOC646762-0.86*ADSS-0.84*TGIF1-0.81*SIAH1-0.83*DET1-0.8*KCTD15-0.79*FCHSD2
LIHC	Score = 0.97*HNRNPH1+0.81*N4BP3+0.82*LDHA+0.81*ZCRB1+0.84*YBX1+0.78*STK39+0.78*ATP6V1E1+0.8*ANXA5+0.78*HN1+0.76*ATP1B3-0.81*STAT5B-0.79*C9orf3-0.79*CHST14-0.76*SIK2-0.72*POLDIP2-0.73*ATF7IP2-0.72*SLC23A2-0.67*STIM1-0.65*MIA3-0.65*PSD4
LUAD	Score = 0.72*ITGA6+0.73*C1QTNF6+0.72*MTHFD1+0.7*DNAJB4+0.7*BACH1+0.69*CCNA2+0.65*EXT1+0.65*FSCN1+0.66*DNAJB6+0.65*NOC3L-0.91*SLC25A42-0.82*PRKCD-0.79*DBP-0.75*DENND1C-0.71*NRL-0.72*C19orf42-0.73*ALAD-0.71*SLC11A2-0.68*ABAT-0.67*FAM117A
LUSC	Score = 0.74*CD14+0.66*ARHGAP1+0.63*CD151+0.62*FSTL3+0.6*RALGAPA2+0.59*CST3+0.57*C11orf2+0.56*SNX29+0.56*FAM109B+0.54*EHD1-0.69*ERH-0.65*NDUFB1-0.59*CBX1-0.56*EMD-0.55*RLIM-0.53*FAM103A1-0.53*MNAT1-0.53*VRK1-0.51*SS18L2-0.5*FKBP3
MESO	Score = 1.71*CDCA8+1.63*KPNA2+1.62*SPAG5+1.54*CCNA2+1.64*IQGAP3+1.66*FOXM1+1.5*HMGB2+1.51*MAD2L1+1.52*CDCA5+1.58*PRC1-1.53*KLHL9-1.44*ETAA1-1.41*THTPA-1.36*HIST1H2BD-1.32*FOXO4-1.39*FBXO44-1.28*HIST1H2AC-1.39*HIST1H2BK-1.3*SH3BGRL-1.36*TMBIM4
OV	Score = 0.6*CBLL1+0.59*CACNA1C+0.56*SOCS5+0.54*ZNF384+0.54*CACNB1+0.53*SEMA4F+0.52*AGPAT6+0.52*CHKA+0.54*GLIS2+0.52*GLCE-0.77*NPEPL1-0.6*TLCD1-0.57*LMO4-0.55*CASP6-0.54*ISG20-0.55*AP4B1-0.53*SAT1-0.52*ZNF326-0.51*ENSA-0.5*AP1S2
PAAD	Score = 1.31*ATG12+1.3*ASCC1+1.33*NFE2L3+1.31*KIAA1609+1.3*CCDC6+1.2*EIF2A+1.26*TMOD3+1.21*AP3S1+1.24*METAP1+1.22*NCK1-1.33*USP20-1.27*MUM1-1.27*REC8-1.24*RBM6-1.21*ARMC5-1.23*DEF8-1.27*KLHL22-1.13*C7orf43-1.14*MGC23284-1.1*ELMOD3
PCPG	Score = 2*GLE1+1.99*EFTUD1+1.99*NARG2+1.98*CIZ1+1.97*ZNF490+1.97*TTC9C+1.96*FAM178A+1.96*ABCA1+1.95*AKAP13+1.95*LOC642852-2.03*HMOX2-1.96*DGCR14-1.96*SLC10A3-1.95*ITFG3-1.94*FAM118A-1.93*MBD3-1.93*USE1-1.92*ICOSLG-1.91*FSCN1-1.91*TMEM167B
PRAD	Score = 20.3*EXTL2+20.3*B3GNT5+20.3*SEMA4C+20.3*NUDCD2+20.3*GNAI1+20.3*THUMPD1+20.3*CNNM3+20.3*RNF138+20.3*PRPF4+20.3*FASTKD3-20.3*MRM1-20.3*DAP-20.3*PAOX-20.3*PLA2G15-20.3*SBNO2-20.3*STK19-20.3*CCDC85C-20.3*TBXAS1-20.3*NFATC1-20.3*HSD17B7
READ	Score = 2.26*PSMA3+2.84*PHLPP1+2.91*CNDP2+2.96*CORO1A+2.2*AKR7A2+2.82*SSBP2+2.82*TMEM173+2.7*ATP6V0C+2.7*NFYC+2.79*B4GALT3-2.28*OSGEPL1-2.96*PHF20-3.01*ANKRD27-2.95*ZNF853-2.95*RAPGEF2-2.88*SETD2-2.87*MSH6-2.85*ATM-3.01*SIRT5-2.81*SGK3
SARC	Score = 1.12*RLIM+1.03*BAIAP3+0.97*FUBP1+1*ZNF146+0.99*ATXN10+0.95*LRRC41+0.93*LRRC47+0.9*DOCK7+0.9*ZNF697+0.89*LAPTM4B-1.13*TRIM21-1.08*B3GALT4-1.04*CCDC69-1.01*CCNDBP1-0.97*C14orf159-0.95*GALK2-0.91*PARP14-0.91*ATP2A3-0.86*C15orf24-0.84*PPAP2A
SKCM	Score = 0.75*HN1L+0.7*GATAD2A+0.68*NT5DC2+0.66*VDAC1+0.65*KPNA2+0.62*FOXM1+0.62*DCTN2+0.61*CDC25A+0.6*SLC25A3+0.61*SLC25A15-0.81*GBP2-0.77*APOL6-0.77*IFITM1-0.75*FCGR2A-0.74*FAM96A-0.72*PARP9-0.72*APOBEC3F-0.71*NXT2-0.7*UBA7-0.7*APOL1
STAD	Score = 2.33*SLC9A3R2+1.91*ITPRIP+1.74*SOCS2+2.04*C1orf144+1.7*LOC282997+1.69*BMP2K+2.64*VPS52+1.93*UBE4B+1.51*CXCR7+1.9*NDUFA11-2.55*SLC33A1-2.27*TMEM66-2.18*UBA5-2.14*CD47-1.8*C21orf59-2.03*NSF-2.01*FUNDC1-1.97*RAB1A-1.69*C14orf142-1.95*PFDN4
TGCT	Score = 1.03*FAM177A1+1.03*NBR2+1*ATAD2B+0.98*C8orf73+0.96*FMNL2+0.95*CEBPA+0.95*VCPIP1+0.94*C12orf23+0.94*LMBR1L+0.94*ABCC5-1.06*MYO1E-1.03*CABLES1-1*FAM84B-0.99*TOP1-0.98*NCSTN-0.97*NAIF1-0.97*IRS2-0.97*HIBADH-0.97*FUBP3-0.97*PGM1
THCA	Score = 2.06*IQSEC1+1.99*FLYWCH1+1.88*ZHX3+2.12*SEMA6A+1.78*FTO+1.76*LARS+1.74*TGFBR3+2.03*PTEN+1.72*ZNF324+2.72*CEP250-1.96*ANXA1-1.89*SEC14L2-2.18*CIR1-2.17*MED17-2.15*ITGB1BP1-1.86*SRP68-2.14*VAMP8-2.08*PSME2-2.77*RPS27-1.73*CLU
THYM	Score = 2.5*RARG+2.45*RBM47+2.39*PELI3+2.39*ATP1B1+2.39*TST+2.35*NUDT16+2.38*DENND1A+2.35*PPAPDC1B+2.34*GNS+2.3*TBC1D16-2.44*ADRBK1-2.43*PDSS1-2.41*SEMA4D-2.4*INTS8-2.4*VRK1-2.4*PTP4A2-2.39*CUTC-2.39*SEMA7A-2.38*SCLT1-2.37*ANKRD27
UCEC	Score = 2.12*TUBB2A+1.92*TAOK3+2.11*ENDOD1+2.05*KLF11+2.06*SYNPO+2.02*BRAF+2.02*SYTL2+2.05*SPAG5+1.72*MCL1+1.73*ARMC1-1.81*SETD6-1.8*LYRM1-1.58*PYCRL-1.58*YDJC-1.71*CRBN-1.94*C15orf29-1.52*PHF5A-2.57*PPA1-1.56*WWOX-1.64*IFT140
UCS	Score = 1.27*S100A10+1.23*PDE4A+1.21*STMN3+1.22*ARL4D+1.18*HIBCH+1.16*FN3K+1.2*SEC23B+1.12*NINJ1+1.16*LOC728554+1.16*CTU1-1.86*CBX5-1.32*DNMT3A-1.31*PSMD7-1.35*PCBP2-1.25*C2orf68-1.18*BUD13-1.17*ZNRF1-1.21*SSRP1-1.19*ST3GAL2-1.22*TUT1
UVM	Score = 2.32*GTF3A+3.14*PSTPIP2+2.27*SPAG1+3.03*SFT2D2+2.23*LIPA+2.2*IMPA1+2.21*JTB+2.16*COQ2+2.93*ALG5+2.97*ISG20-3.14*RABL2B-2.26*C16orf86-2.19*CNP-3.01*C3orf39-2.19*C3orf37-2.17*TBKBP1-2.14*TOM1L2-2.17*RPL32P3-2.89*PPP2R3B-2.16*QRICH1

To assess the clinical utility of the risk score, correlation of the risk score with the clinical variables in the training group was explored. In the analysis, higher scores were associated with male patients, patients with older age, and patients with advanced tumor stages (Figure 2C). Further cox analysis and log rank test also confirmed that the poor survival outcome in patients with higher risk scores in different tumor stages (Figure 2D–2E). In the testing group, similar relationship between the risk score and clinical variables was also shown (Figure 2F). Notably, with respect to survival analysis, higher risk scores in the testing group also indicated higher risks of prognosis, suggesting that the risk score showed valuable clinical utility (Figure 2G–2H).

### Evaluation of the prognostic genes and risk scores

The RNA-seq data demonstrated great value for cancer prognosis. Risk scores specific for each type of cancer were shown in Table 1, which were calculated by applying the method used in the whole cancer population. However, at this moment, these prognostic models are limited by the sample size to be of clinical value.

To evaluate the effect of different normalization method for the RNA-seq data, quantile data were transformed into z-score or being applied the voom normalization. As shown in supplementary Figure 1A-1B, after quantile normalization of the RNA-seq data, the z-score transformation or voom normalization doesn't change much of the prognostic genes (based on z values of cox regression), with the pearson r value of 0.98 and 0.97, respectively. After various normalization method, top prognostic genes remained mostly the same, which was shown in the supplementary Figure 1C. Thus, applying Z-score transformation or voom normalization yield limited value for the survival analysis.

For the above prognostic model, the high or low expression for prognostic model was determined by the median expression level of each gene. The gene was divided as binary categorization such as 1 for high expression (> median value) and 0 for low expression (< median value). Here we also applied the z-score (continuous variable) directly to propose a prognostic model that can reflect the values of gene expression (Table 1). Cox regression results showed that the continuous prognostic model have an hazard ratio of 1.22, which means the death risk increases by 22% if the patients get a risk score increased by 1.

The prognostic genes (in each cancer type and in the whole cancer population) were filtered by a specific cutoff (|z| > 3.09, or nominal one-sided p < 0.001). As an investigation of the relationship of different prognostic gene across different cancer types, prognostic genes in each cancer type were compared with the whole cancer population. As shown in the supplementary Figure 1D, for the prognostic gene identified in the study on single cancer type, most of them were also found in the pooled analysis. For example, 66% of the prognostic genes in the ACC also had prognostic values in the whole cancer population.

### Pathway analysis in patients with different prognosis

**Figure 3:**
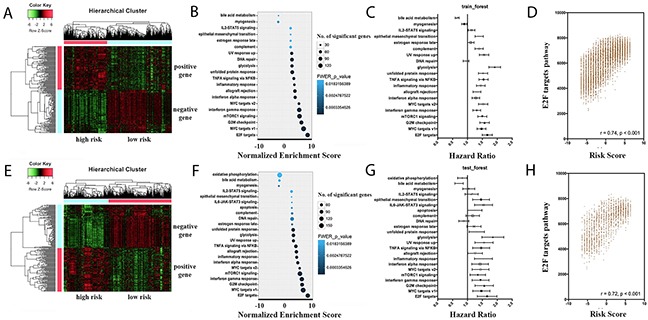
Prognostic landscape of pathway scores in the whole cancer population **A, E.** Heatmap depicting gene expression levels after unsupervised hierarchical clustering in the training set and testing set, respectively. Expression levels are indicated on a low-to-high scale (green-black-red). Two clusters are defined, namely the high risk group and low risk group. **B, F.** GSEA analysis was performed in the training set and testing set, respectively, to identify biological pathways associated with survival outcome. FWER-p values are indicated on a low-to high scale (lightblue-darkblue). The number of significant genes in each gene set is indicated by the circle size. **C, G.** Forest plots of pathway score association with cancer mortality in the training set and testing set, respectively. **D, H.** Scatter plots of correlations between risk scores and the E2F pathway scores in the training set and testing set, respectively.

Based on the prognostic risk score, patients were stratified into two different survival groups of a positive risk score and a negative risk score. This unsupervised cluster analysis showed obvious distinctions between the stratified survival groups, both in the training group and the testing group (Figure 3A, 3E). To link the observed gene expression changes with molecular pathways that may impact the differential survival between high- and low-risk groups, gene set enrichment analysis (GSEA) was performed. As shown in Figure 3B and 3F, pathways such as E2F targets, MYC targets, G2M checkpoint, mTORC1 signaling and interferon gamma response were significantly enriched in the patients of higher risk scores, with good consistency between the training group and the testing group.

In order to assess possible effects of different pathways, the GSEA for every sample were evaluated using the single sample gene set enrichment analysis (ssGSEA). Based on the calculated scores for each pathway, cox analysis was performed to evaluate their prognostic effects. Results showed that most of the significant pathways from the GSEA output showed positive correlations with the survival outcome (Figure 3C, 3G). In addition to the cox analysis, positive correlations were detected between the pathway ssGSEA scores and the prognostic risk scores. In Figure 3D and 3H, correlation analysis were shown in the most significant pathway (E2F targets), in both the training group and testing group.

### Assessment of prognostic power of gene expression data

**Figure 4:**
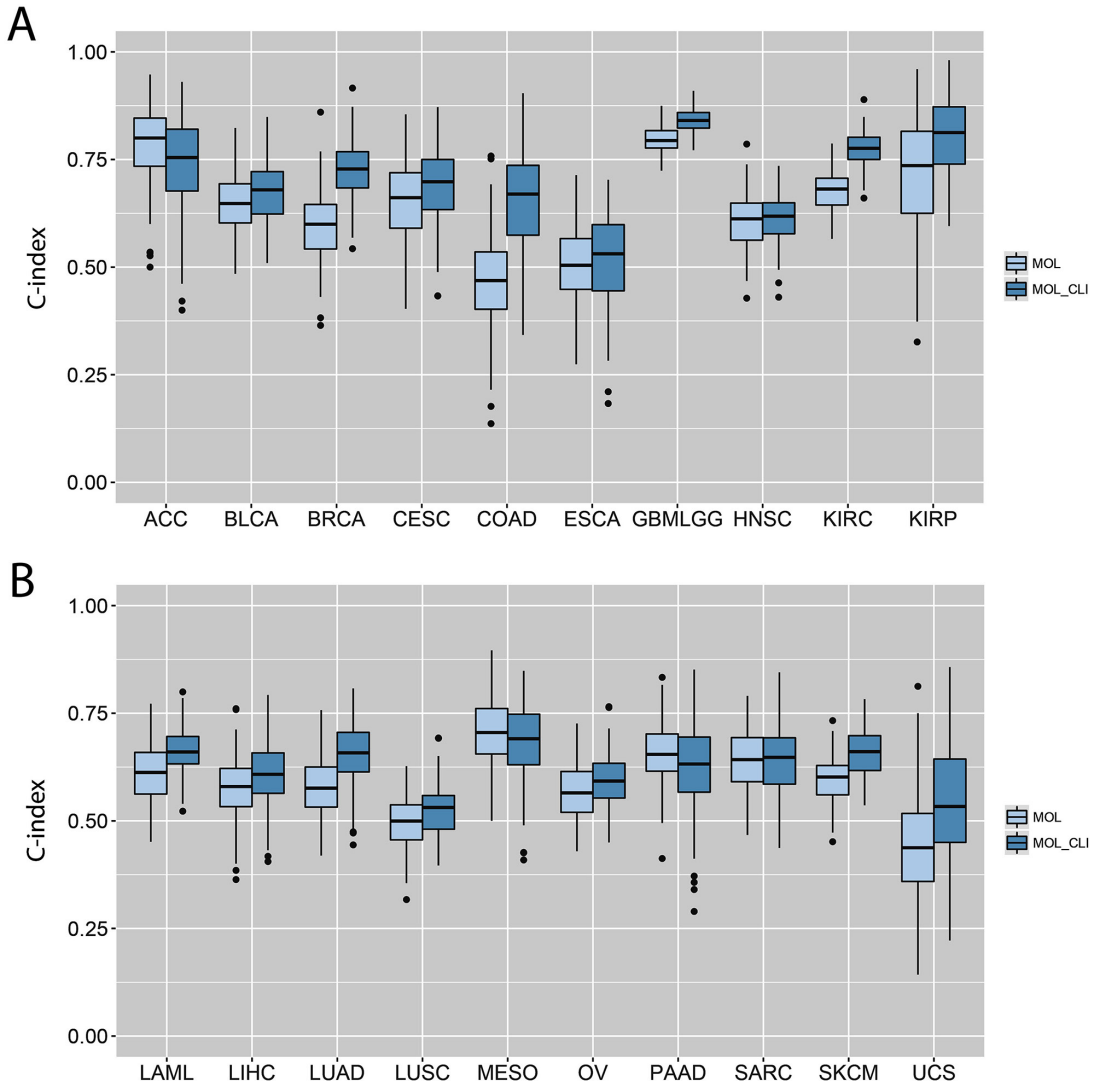
C-indexes by models trained from individual gene expression data alone or in combination with clinical variables **A.** C-indexes calculated from the ACC, BLCA, BRCA, CESC, COAD, ESCA, GBMLGG, HNSC, KIRC and KIRP. **B.** C-indexes calculated from the LAML, LIHC, LUAD, LUSC, MESO, OV, PAAD, SARC, SKCM and UCS. The lightblue box indicates the model built from individual gene expression data alone, and the darkblue box indicates the model built from the combination of gene expression data and clinical variables.

Since the gene expression analysis and pathway analysis showed great prognostic values in the study, prognostic power of gene expression data were further explored. C-index was applied to assess the predictive power of the gene expression data alone or combined with clinical information. To improve accuracy, cancer types that don't have enough death events (< 20 deaths or < 10% mortality) were excluded. Cancer patients were randomly split into 80% training and 20% testing for 100 times to calculate the final C-index. As shown in Figure 4A and 4B, the predictive power of gene expression data alone varied across cancer types. In KIRC and GBMLGG, the prognostic power was much higher when compared with other cancer types.

To explore any additional prognostic power, the gene expression data was combined with clinical information. Significant clinical features (correlated with survival) were applied as baseline to build the cox model. A feature-selection step against the residuals was utilized to include the gene features that better fit the model. Results showed that the most gene expression data alone (18 out of 20 cases) had significant predictive power (C-index > 0.5). Incorporating clinical information to gene expression data statistically boosts the model performance in 12 cancer types (BLCA, BRCA, CESC, GBMLGG, KIRC, KIRP, LAML, LIHC, LUAD, LUSC, OV, SKCM) (p < 0.05) (Figure 4A, 4B).

## DISCUSSION

In this study, we assessed the clinical utility of genomic expression data from ~9000 cancer patients of 32 tumor types. The prognostic power across different cancer types was also evaluated [7, 8].

Currently, only a few gene expression-based markers are routinely used in clinical practice [9–12]. The clinical utility of genomic expression has not been fully explored. Yuan *et al*. reported that for cancer patients, incorporating molecular features with clinical information yields significantly improved predictions. However, they only focused on 4 cancer types (KIRC, GBM, OV, LUSC), and no conclusions could be drawn for the whole cancer population [13]. Recently, Gentles *et al*. described the genomic prognostic landscape across human cancers, highlighting the promise of genomic expression data as biomarkers for clinical outcomes [14]. In our study, besides illuminating the prognostic landscape of genomic expression, pathway analysis based on these prognostic genes was also evaluated. In addition, the C-index was calculated from the prognostic models across tumor types, to assess the prognostic power of gene expression data.

Based on the genomic expression data in the whole cancer population, the top prognostic genes were identified, such as FOXM1, CBX7, CREBL2 and SKP2, which were consistent with previous studies [14–16]. Notably, when building the risk scores based on these top prognostic genes, significant stratification in survival outcomes were shown, both in the training and validation cohorts, indicating the robustness of the predicting effect of the prognostic genes.

Because of heterogeneity, many statistical methods have been developed to analyze cancer genomics, based on gene sets, pathways and network modules [17–19]. For the first time, our study described the prognostic landscape of biological pathways in the whole cancer population. Gene set enrichment of the differentially expressed genes revealed significant prognostic pathways, such as the E2F targets, MYC targets, G2M checkpoint, interferon gamma response, and so on. Mostly, these pathways are correlated with cell cycle, proliferation and inflammation, which is consistent with the biological mechanisms of tumor progression [20, 21].

To explore prognostic power, our results showed that combining the clinical and molecular information could improve the predictive power of the gene expression data in most cancer types. Although the absolute magnitude gains were limited, the gene-expression signatures provide new biological insights into the process of cancer progression and metastasis that can help to improve the prediction power [22]. Actually, some of the gene-based prognostic signatures have already been demonstrated to be clinically useful for predicting the risk of tumor recurrence, such as the 70-gene and 76-gene signatures in breast cancer [23–26].

It is also important to realize that gene expression information is just one of the abundant molecular data (genomic, transcriptomic, epigenomic and proteomic) revealing the biological complexity of cancer. Other molecular information will also improve our understanding of the genotype–phenotype relationships involved in cancer. On the other hand, regarding the reliability and the reproducibility of the clinical use of molecular data, future technology, statistical and analytical methods are in great need to catch up with clinical needs [22].

In conclusion, our gene analysis and pathway analysis showed significant values for the prediction of survival outcomes for cancer patients. Additionally, it was found that by combining clinical information with molecular data, the model performance could be boosted statistically in most cancer types. However, further efforts would be needed to generate prognostic models ready for clinical use in the future.

## MATERIALS AND METHODS

### Data set compilation

Clinical and survival data were acquired from the TCGA Data Portal (https://tcga-data.nci.nih.gov/tcga/). RNA sequencing data was obtained from the GDAC Firehose System (http://gdac.broadinstitute.org/). To maintain data consistency, only the RNA sequencing data from the platform of Illumina HiSeq 2000 RNA Sequencing V2 was included. Patients who have a complete clinical and RNA sequencing data were screened for further analysis. For each cancer data set, patients were split into two groups randomly: 80% as the training set and 20% as the testing set. For the pan-cancer study, all RNA sequencing data were combined by intersecting the common genes across different cancer types.

### Prognostic genes and construction of the prognostic model

For RNA sequencing data, all “raw count” values were divided by the 75th percentile of the same patient (after removing zeros) and multiplied by 1000, to get the quantile normalization for survival analysis. Furthermore, quantile data were also transformed to the z-score or normalized by “voom” to evaluate the effects of different normalization method. The Z-score was calculated as “(tumor expression - mean expression in reference) / standard deviation of expression in reference”. The voom normalization was applied using the R package “limma”. It estimates the mean-variance relationship of the log-counts and generates a precision weight for each observation.

The association of each gene expression with survival outcomes was assessed via cox proportional hazards regression using the ‘coxph’ function of the R ‘survival’ package. Cox coefficients, hazard ratios with 95% confidence intervals, p values, and z-scores were obtained for each array probe. Top prognostic genes were identified by the values of z-scores. Based on these top prognostic genes, risk scores were built and it was defined as the weighted sums of the independent prognostic gene values (1 for high expression, and 0 for low expression). They were weighted with their regression coefficients from the cox models. Based on the prognostic risk score, further cox regression analysis and correlation analysis with clinical variables were performed.

### Differential expression analysis and clustering analysis

Differential expression analysis was done using R “limma” package. Based on the limma output for the most differentially expressed genes, unsupervised hierarchical clustering analysis was used to discover the gene expression patterns of these groups sharing common characteristics. Heatmap was constructed using the R “gplots” package.

### Gene set enrichment analysis

Prognostic gene sets are groups of genes that share common biological function. The evaluation of prognostic gene sets was performed using gene set enrichment analysis (GSEA) [27], where gene sets were obtained from the Molecular Signatures Database (mSigDB) [28]. In addition, a variant of GSEA, termed single sample gene set enrichment analysis (ssGSEA) was applied to calculate separate enrichment scores for each pairing of a sample and gene set [29]. Further cox regression analysis and correlation analysis were performed based on the enrichment scores of each gene set.

### Performance evaluation of gene expression data

Performance evaluation of gene expression data was conducted based on the method suggested by Yuan *et al* [13]. Firstly, univariate cox was applied to the training set to select the top features correlated with survival, which were then converged by the LASSO using the R package “glmnet”. The model was then applied to the testing set for prediction. Concordance index (C-index) was estimated from 100 randomizations using the R package “survcomp”. To explore the predictive power of integrating gene expression data with clinical information, we used the significant clinical features (correlated with survival) as baseline to build the cox model. Then a feature-selection step against the residuals was applied to combine the gene features that better fit the model.

## SUPPLEMENTARY FIGURE



## References

[1] Siegel RL, Miller KD, Jemal A (2016). Cancer statistics, 2016. CA Cancer J Clin.

[2] Van Allen EM, Wagle N, Levy MA (2013). Clinical analysis and interpretation of cancer genome data. Journal of clinical oncology.

[3] Mansouri L, Gunnarsson R, Sutton LA, Ameur A, Hooper SD, Mayrhofer M, Juliusson G, Isaksson A, Gyllensten U, Rosenquist R (2012). Next generation RNA-sequencing in prognostic subsets of chronic lymphocytic leukemia. American journal of hematology.

[4] Tang H, Xiao G, Behrens C, Schiller J, Allen J, Chow CW, Suraokar M, Corvalan A, Mao J, White MA, Wistuba II, Minna JD, Xie Y (2013). A 12-gene set predicts survival benefits from adjuvant chemotherapy in non-small cell lung cancer patients. Clinical cancer research.

[5] Chia SK, Bramwell VH, Tu D, Shepherd LE, Jiang S, Vickery T, Mardis E, Leung S, Ung K, Pritchard KI, Parker JS, Bernard PS, Perou CM, Ellis MJ, Nielsen TO (2012). A 50-gene intrinsic subtype classifier for prognosis and prediction of benefit from adjuvant tamoxifen. Clinical cancer research.

[6] Weinstein JN, Collisson EA, Mills GB, Shaw KR, Ozenberger BA, Ellrott K, Shmulevich I, Sander C, Stuart JM, Cancer Genome Atlas Research N (2013). The Cancer Genome Atlas Pan-Cancer analysis project. Nature genetics.

[7] Cristescu R, Lee J, Nebozhyn M, Kim KM, Ting JC, Wong SS, Liu J, Yue YG, Wang J, Yu K, Ye XS, Do IG, Liu S (2015). Molecular analysis of gastric cancer identifies subtypes associated with distinct clinical outcomes. Nature medicine.

[8] De Sousa EMF, Wang X, Jansen M, Fessler E, Trinh A, de Rooij LP, de Jong JH, de Boer OJ, van Leersum R, Bijlsma MF, Rodermond H, van der Heijden M, van Noesel CJ (2013). Poor-prognosis colon cancer is defined by a molecularly distinct subtype and develops from serrated precursor lesions. Nature medicine.

[9] Arteaga CL, Sliwkowski MX, Osborne CK, Perez EA, Puglisi F, Gianni L (2012). Treatment of HER2-positive breast cancer: current status and future perspectives. Nature reviews Clinical oncology.

[10] Dowsett M, Nielsen TO, A'Hern R, Bartlett J, Coombes RC, Cuzick J, Ellis M, Henry NL, Hugh JC, Lively T, McShane L, Paik S, Penault-Llorca F (2011). Assessment of Ki67 in breast cancer: recommendations from the International Ki67 in Breast Cancer working group. Journal of the National Cancer Institute.

[11] Ludwig JA, Weinstein JN (2005). Biomarkers in cancer staging, prognosis and treatment selection. Nature reviews Cancer.

[12] Sidransky D (2002). Emerging molecular markers of cancer. Nature reviews Cancer.

[13] Yuan Y, Van Allen EM, Omberg L, Wagle N, Amin-Mansour A, Sokolov A, Byers LA, Xu Y, Hess KR, Diao L, Han L, Huang X, Lawrence MS (2014). Assessing the clinical utility of cancer genomic and proteomic data across tumor types. Nature biotechnology.

[14] Gentles AJ, Newman AM, Liu CL, Bratman SV, Feng W, Kim D, Nair VS, Xu Y, Khuong A, Hoang CD, Diehn M, West RB, Plevritis SK, Alizadeh AA (2015). The prognostic landscape of genes and infiltrating immune cells across human cancers. Nature medicine.

[15] van 't Veer LJ, Dai H, van de Vijver MJ, He YD, Hart AA, Mao M, Peterse HL, van der Kooy K, Marton MJ, Witteveen AT, Schreiber GJ, Kerkhoven RM, Roberts C (2002). Gene expression profiling predicts clinical outcome of breast cancer. Nature.

[16] Chan CH, Li CF, Yang WL, Gao Y, Lee SW, Feng Z, Huang HY, Tsai KK, Flores LG, Shao Y, Hazle JD, Yu D, Wei W (2012). The Skp2-SCF E3 ligase regulates Akt ubiquitination, glycolysis, herceptin sensitivity, and tumorigenesis. Cell.

[17] Ma S, Kosorok MR, Huang J, Dai Y (2011). Incorporating higher-order representative features improves prediction in network-based cancer prognosis analysis. BMC medical genomics.

[18] Lee E, Chuang HY, Kim JW, Ideker T, Lee D (2008). Inferring pathway activity toward precise disease classification. PLoS computational biology.

[19] Goeman JJ, Buhlmann P (2007). Analyzing gene expression data in terms of gene sets: methodological issues. Bioinformatics.

[20] Tachibana KE, Gonzalez MA, Coleman N (2005). Cell-cycle-dependent regulation of DNA replication and its relevance to cancer pathology. The Journal of pathology.

[21] Coussens LM, Werb Z (2002). Inflammation and cancer. Nature.

[22] Sotiriou C, Piccart MJ (2007). Taking gene-expression profiling to the clinic: when will molecular signatures become relevant to patient care?. Nature reviews Cancer.

[23] Bueno-de-Mesquita JM, van Harten WH, Retel VP, van't Veer LJ, van Dam FS, Karsenberg K, Douma KF, van Tinteren H, Peterse JL, Wesseling J, Wu TS, Atsma D, Rutgers EJ (2007). Use of 70-gene signature to predict prognosis of patients with node-negative breast cancer: a prospective community-based feasibility study (RASTER). The Lancet Oncology.

[24] Buyse M, Loi S, van't Veer L, Viale G, Delorenzi M, Glas AM, d'Assignies MS, Bergh J, Lidereau R, Ellis P, Harris A, Bogaerts J, Therasse P (2006). Validation and clinical utility of a 70-gene prognostic signature for women with node-negative breast cancer. Journal of the National Cancer Institute.

[25] Wang Y, Klijn JG, Zhang Y, Sieuwerts AM, Look MP, Yang F, Talantov D, Timmermans M, Meijer-van Gelder ME, Yu J, Jatkoe T, Berns EM, Atkins D (2005). Gene-expression profiles to predict distant metastasis of lymph-node-negative primary breast cancer. Lancet.

[26] Desmedt C, Piette F, Loi S, Wang Y, Lallemand F, Haibe-Kains B, Viale G, Delorenzi M, Zhang Y, d'Assignies MS, Bergh J, Lidereau R, Ellis P (2007). Strong time dependence of the 76-gene prognostic signature for node-negative breast cancer patients in the TRANSBIG multicenter independent validation series. Clinical cancer research.

[27] Subramanian A, Tamayo P, Mootha VK, Mukherjee S, Ebert BL, Gillette MA, Paulovich A, Pomeroy SL, Golub TR, Lander ES, Mesirov JP (2005). Gene set enrichment analysis: a knowledge-based approach for interpreting genome-wide expression profiles. Proceedings of the National Academy of Sciences of the United States of America.

[28] Liberzon A, Subramanian A, Pinchback R, Thorvaldsdottir H, Tamayo P, Mesirov JP (2011). Molecular signatures database (MSigDB) 3. 0. Bioinformatics.

[29] Barbie DA, Tamayo P, Boehm JS, Kim SY, Moody SE, Dunn IF, Schinzel AC, Sandy P, Meylan E, Scholl C, Frohling S, Chan EM, Sos ML (2009). Systematic RNA interference reveals that oncogenic KRAS-driven cancers require TBK1. Nature.

