# Supplementary material for: Assessing the clinical utility of genomic expression data across human cancers
Source: Oncotarget. 2016 Jun 14;7(29):45926–36. doi: 10.18632/oncotarget.10002 (PMC5216771; doi:10.18632/oncotarget.10002)
Supplement: Supplementary file 1 [file oncotarget-07-45926-s001.pdf]

Assessing the clinical utility of genomic expression data across human cancers

SUPPLEMENTARY FIGURE

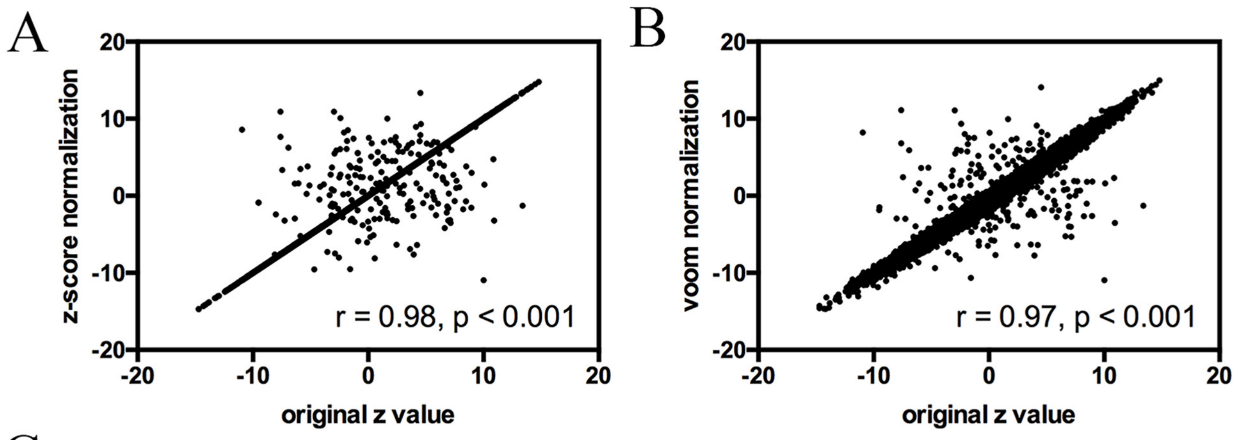

C

Original	positive	B3GNT5 SLC11A1 ELF4 GALNT2 PA2G4P4 SKP2 S100A9 FOXM1 PSMB2 ARL6IP6
	negative	GPD1L SECISBP2 XPC TMED8 CREBL2 CRY2 MAGED2 CIRBP CBX7 TADA2B
Z-score normalize	positive	B3GNT5 SLC11A1 ELF4 GALNT2 PA2G4P4 SKP2 S100A9 FOXM1 PSMB2 ARL6IP6
	negative	GPD1L SECISBP2 XPC TMED8 CREBL2 CRY2 MAGED2 CIRBP CBX7 TADA2B
Voom normalize	positive	B3GNT5 SLC11A1 GALNT2 PSMB3 PA2G4P4 SLC2A1 SKP2 S100A9 IRAK1 ELF4
	negative	CRY2 COQ7 XPC FBXW4 TMED8 TADA2B CREBL2 CBX7 CIRBP MAGED2

D

ACC(66%), BLCA(41%), BRCA(49%), CESC(61%), CHOL(100%), COAD(100%), ESCA(62%), GBMLGG(56%)
HNSC(52%), KIRC(57%), KIRP(85%), LAML(47%), LIHC(59%), LUAD(76%), LUSC(39%), MESO(74%)
OV(35%), PAAD(57%), SARC(66%), SKCM(56%), THCA(100%), UCEC(100%), UCS(43%)

Supplementary Figure S1: Evaluation of the prognostic genes and risk scores. A. Scatterplot of the cox regression results based on the original quantile RNA-seq data and z-score normalization RNA-seq data. **B.** Scatterplot of the cox regression results based on the original quantile RNA-seq data and voom normalization RNA-seq data. **C.** Top adversely and favorably prognostic genes calculated from the RNA-seq data using different normalization method (original quantile normalization, z-score normalization and voom normalization). **D.** Percentage of the significant prognostic genes shared by each cancer type with the whole cancer population.
